# *Eggerthella timonensis* sp. nov, a new species isolated from the stool sample of a pygmy female

**DOI:** 10.1002/mbo3.575

**Published:** 2018-06-13

**Authors:** Melhem Bilen, Maxime Descartes Mbogning Fonkou, Enora Tomei, Nicholas Armstrong, Fadi Bittar, Jean‐Christophe Lagier, Ziad Daoud, Pierre‐Edouard Fournier, Didier Raoult, Frédéric Cadoret

**Affiliations:** ^1^ URMITE UM63 CNRS 7278 IRD 198 INSERM 1095 Institut Hospitalo‐Universitaire Méditerranée‐Infection Faculté de médecine Aix‐Marseille Université Marseille Cedex France; ^2^ Clinical Microbiology Department Faculty of Medicine and Medical sciences University of Balamand Amioun Lebanon; ^3^ Special Infectious Agents Unit King Fahd Medical Research Center King Abdulaziz University Jeddah Saudi Arabia

**Keywords:** culturomics, *Eggerthella timonensis*, genome, new species, pygmy, taxonogenomics

## Abstract

*Eggerthella timonensis* strain Marseille‐P3135 is a new bacterial species, isolated from the stool sample of a healthy 8‐year‐old pygmy female. This strain (LT598568) showed a 16S rRNA sequence similarity of 96.95% with its phylogenetically closest species with standing in nomenclature *Eggerthella lenta* strain DSM 2243 (AF292375). This bacterium is a nonspore forming, Gram‐positive, nonmotile rod with catalase but no oxidase activity. Its genome is 3,916,897 bp long with 65.17 mol% of G + C content. Of the 3,371 predicted genes, 57 were RNAs and 3,314 were protein‐coding genes. Here, we report the main phenotypic, biochemical, and genotypic characteristics of *E. timonensis* strain Marseille‐P3135 (=CSUR P3135, =CCUG 70327); ti.mo.nen′sis, N.L. masc. adj., with *timonensis* referring to La Timone, which is the name of the hospital in Marseille (France) where this work was performed). Strain is a nonmotile Gram‐positive rod, unable to sporulate, oxidase negative, and catalase positive. It grows under anaerobic conditions between 25°C and 42°C but optimally at 37°C.

## 1 INTRODUCTION

The human gut microbiota has drawn more attention to with the advancement and development of new sequencing techniques (Gill et al., 2006; Ley, Turnbaugh, Klein, & Gordon, 2006; Ley et al., 2005). Yet, we face several limitations when using these techniques, especially when it comes to depth bias, incomplete database, and the obtention of raw material for further analysis (Greub, 2012). However, the ability to cultivate and isolate pure colonies is mandatory to describe the human gut microbiota, thus the need to develop a technique that enhances the efficiency of these two factors (Lagier et al., 2015). When talking about the human gut, stool samples are the best representatives of its microbiome since only 1 g of human stool sample might contain up to 10^11^–10^12^ bacteria (Raoult & Henrissat, 2014). Before the introduction of culturomics, only 688 bacteria and 2 archaea had been recognized in the human gut (Lagier et al., 2016). Culturomics was developed with the purpose of optimizing growth conditions of previously uncultured bacteria in order to fill the missing gaps in the human microbiome (Lagier et al., 2012a). In general, culturomics consists in culturing samples by using 18 different conditions along with isolating pure colonies for further identifications using the matrix‐assisted laser desorption/ionization time‐of‐flight mass spectrometry (matrix‐assisted laser desorption/ionization time‐of‐flight mass spectrometry [MALDI‐TOF MS]) approach and 16S rRNA gene sequencing. Any un‐identified colonies are subject to 16S rRNA gene sequencing and a series of descriptive experiments targeting the phenotypic, biochemical, and genomic characteristics at the same time (Lagier et al., 2012b; Seng et al., 2009). Using this methodology, we were able to isolate a new strain *Eggerthella timonensis*, a member of the genus *Eggerthella* (Bilen, Cadoret, Daoud, Fournier, & Raoult, 2016). *Eggerthella lenta*, formerly known as *Eubacterium lentum*, is the type strain of *Eggerthella* genus and was first reported in 1935 by Arnold Eggerth (Eggerth, 1935; Kageyama, Benno, & Nakase, 1999; Moore, Cato, & Holdeman, 1971). Species belongs to *Eggerthella* genus, *Actinobacteria* phylum in the *Coriobacteriaceae* family and known for its ability to grow under anaerobic conditions (Kageyama et al., 1999). Moreover, *Eggerthella* species have been reported to colonize the human gut microbiome and have been correlated to several health problems such as anal abscess and ulcerative colitis (Lau et al., 2004a).

In this study, we describe *E. timonensis* strain Marseille‐P3135 (=CCUG 70327 [Culture Collection University Gothenburg], =CSUR P3135 [Collection de Souches de l'Unité des Rickettsies]) using a polyphasic approach by targeting multiple phenotypic, biochemical, and genotypic aspects.

## 2 MATERIAL AND METHODS

### 2.1 Strain isolation

Before stool sample collection in Congo in 2015, an approval was obtained from the ethic committee (09‐022) of the Institut Hospitalo‐Universitaire Méditerranée Infection (Marseille, France). The stool sample was collected from a healthy 8‐year‐old pygmy female accordingly to Nagoya protocol. Stool samples were shipped from Congo to France in the specific protecting medium C‐Top Ae‐Ana (Culture Top, Marseille, France) and stored at −80°C for further study and analysis.

Samples were inoculated in a blood culture bottle (BD BACTEC^®^, Plus Anaerobic/F Media, Le Pont de Claix, France) supplemented with 5% of rumen and 5% of sheep blood at 37°C. Bacterial growth and isolation was assessed during 30 days on 5% sheep blood–enriched Columbia agar solid medium (bioMérieux, Marcy l'Etoile, France). MALDI‐TOF MS was used for colonies identification. When the latter fails to identify tested colonies, 16S rRNA gene sequencing was used (Lagier et al., 2012b; Seng et al., 2009). On average, 10,000 colonies have been tested for each stool sample.

### 2.2 MALDI‐TOF MS and 16S rRNA gene sequencing

Using a MSP 96 MALDI‐TOF target plate, bacterial colonies were spotted and identified by the means of MALDI‐TOF MS using a Microflex LT spectrometer as previously described (Seng et al., 2009). In case of MALDI‐TOF's identification failure due to lack of a reference strain in the database, 16S rRNA sequencing was used for further analysis using the GeneAmp PCR System 2720 thermal cyclers (Applied Biosystems, Foster City, CA, USA) and the ABI Prism 3130xl Genetic Analyzer capillary sequencer (Applied Biosystems) (Morel et al., 2015). Sequences were assembled and modified using CodonCode Aligner software (http://www.codoncode.com) and finally blasted against the online database of National Center for Biotechnology Information (NCBI) database (http://blast.ncbi.nlm.nih.gov.gate1.inist.fr/Blast.cgi). Once blasted, a sequence similarity of less than 98.65% with the closest species was used to define a new species and 95% for defining a new genus (Kim, Oh, Park, & Chun, 2014). Subsequently, the mass spectrum of the new species was added to the URMITE [Unité de Recherche sur les Maladies Infectieuses et Tropicales Emergentes] database (http://www.mediterranee-infection.com/article.php?laref=256&titre=urms-database) and its 16S rRNA gene sequence was submitted to EMBL‐EBI with an accession number of LT598568.

### 2.3 Phylogenetic analysis

16S rRNA sequences of strain's closest species were obtained from the database of “The All‐Species Living Tree” Project of Silva (LTPs121) (“The SILVA and ‘All‐species Living Tree Project (LTP)’ taxonomic frameworks,” n.d.), aligned with Muscle software and phylogenetic inferences were done using FastTree with the approximately maximum‐likelihood method (Price, Dehal, & Arkin, 2009). Moreover, Shimodaira–Hasegawa test was adapted in order to compute the support local values shown on the nodes. Bad taxonomic reference strains were removed along with duplicates using phylopattern (Gouret, Thompson, & Pontarotti, 2009). This pipeline was done using the DAGOBAH software (Gouret et al., 2011), which comprises Figenix (Gouret et al., 2005) libraries.

### 2.4 Growth conditions

In order to obtain the optimal growth conditions, the strain was cultured under several conditions in terms of temperature, atmosphere, pH, and salinity. First, the strain was cultured and incubated under aerobic, anaerobic, and micro‐aerophilic conditions on 5% sheep blood–enriched Colombia agar (bioMérieux) at the following temperatures: 28°C, 37°C, 45°C, and 55°C. Bacterial growth under anaerobic and microaerophilic environment was tested using the GENbag anaer and GENbag microaer systems (Thermofisher Scientific, Basingstoke), respectively. Furthermore, salinity tolerance was tested by assessing growth at 37°C under anaerobic condition using different NaCl concentrations (0, 5, 10, 50, 75, and 100 g/L NaCl). As well, optimal pH for growth was evaluated by testing multiple pH: 6, 6,5, 7, and 8.5

### 2.5 Morphological and biochemical assays

In order to biochemically describe strain Marseille‐P3135; different API tests (ZYM, 20A and 50CH, bioMérieux) were used. Sporulation ability of this bacterium was tested by exposing a bacterial suspension for 10 min to a thermal shock at 80°C, and then cultured on COS media. Moreover, the motility of the strain was detected using a DM1000 photonic microscope (Leica Microsystems, Nanterre, France) under a 100× objective lens. Also, a bacterial suspension was fixed with a solution of 2.5% glutaraldehyde in 0.1 mol/L cacodylate buffer for more than 1 hr at 4°C for observation under the Morgagni 268D (Philips) transmission electron microscope. Finally, Gram staining results and images were obtained by DM1000 photonic microscope (Leica Microsystems) using a 100× oil‐immersion objective lens.

### 2.6 Fatty acid methyl ester (FAME) composition of strain Marseille‐P3135

Using gas chromatography/mass spectrometry (GC/MS), Cellular FAME analysis was performed. Harvested from several culture plates, two samples were made with <1 mg of bacterial biomass per tube, and then FAME and GC/MS were done as previously described (Dione et al., 2016).

### 2.7 Antibiotic susceptibility

An antibiotic resistance profile was developed by the use of the E test method. The following antibiotics strips were used: vancomycin, rifampicin, benzylpenicillin, amoxycillin, imipenem, tigecycline, amikacin, erythromycin, minocycline, teicoplanin, colistin, daptomycin, metronidazole, and ceftriaxone.

### 2.8 DNA extraction and genome sequencing

To extract the genomic DNA (gDNA) of strain Marseille‐P3135, FastPrep BIO 101 (Qbiogene, Strasbourg, France) was used for a mechanical treatment with acid‐washed beads (G4649‐500 g Sigma). Then, samples were incubated with lysozyme after 2 hr and a half at 37°C and EZ1 biorobot (Qiagen) was used for DNA extraction according the to manufacture guidelines. Qubit was used for DNA quantification (69.3 ng/μl).

As for genome sequencing, MiSeq Technology (Illumina Inc, San Diego, CA, USA) was used with mate‐pair and paired‐end methods. Also, Nextera XT kit (Illumina) and Nextera Mater pair kit (Illumina) were used for samples barcoding. The DNA of the strain was mixed with 11 paired‐end projects and 11 mate‐pair projects. Pair‐end libraries were prepared by using 1 ng of gDNA, which was fragmented and tagged. Twelve PCR amplification cycles accomplished the tag adapters and added dual‐index barcodes. Subsequently, purification was done using AMPure XP bead (Beckman Coulter Inc, Fullerton, CA, USA), and libraries’ normalization was done as described in Nextera XT protocol (Illumina) for pooling and sequencing on MiSeq. A single run of 39 hr in 2 × 250‐bp was done for paired‐end sequencing and clusters generation. This library was loaded on two flowcells. Total information of 6.5 and 4.3 Gb was obtained from a 685 and 446 k/mm^2^ cluster density with a cluster quality 95.1% and 94.8% (12,615,000 and 8,234,000 passed filtered clusters). Index representation for strain Marseille‐P3135 was determined to be of 4.57% and 3.83%. The 576,647 and 315,481 paired‐end reads were filtered based on the quality of the reads.

As for mate‐pair libraries preparation, 1.5 μg of gDNA were used with Nextera mate‐pair Illumina protocol. Mate‐pair junction adapter were used to tag fragmented gDNA, and Agilent 2100 BioAnalyzer (Agilent Technologies Inc, Santa Clara, CA, USA) was used to validate the profile of the fragmentation on a DNA 7500 labchip. The average size of the fragmented DNA ranged between 1 kb up to 11 kb with an ideal size at 9.1 kb. Six hundred nanograms of tagmented DNA (with no size selection) was circularized and sheared mechanically on Covaris device S2 in microtubes (Covaris, Woburn, MA, USA) to reach a size of 956 bp. Then, libraries concentration was set to 8.46 nmol/L, and High Sensitivity Bioanalyzer LabChip (Agilent Technologies Inc, Santa Clara, CA, USA) was used for libraries profile visualization. This library was loaded on two flowcells. Before pooling the libraries, it was normalized at 2 nmol/L. Samples were denatured and diluted at 15 pmol/L before loading it on a the reagent cartridge and then onto the instrument along with the flow cell. A single run of 39 hr was done for cluster generation and sequencing in a 2 × 151‐bp. 5.1 and 2.8 Gb 2.8 were obtained from 542 and 274 K/mm^2^ cluster density with a cluster threshold of 95.7% and 97.6% (10,171,000 and 5,537,000 passing filtered paired reads). Index representation of the studied strain was determined to be of 8.53% and 9.24%. The 867,401 and 511,563 paired reads were trimmed and then assembled.

Genome assembly, annotation, and comparison were made with the same pipeline as previously discussed in our previous work (Elsawi et al., 2017).

## 3 RESULTS AND DISCUSSION

### 3.1 Strain Marseille‐P3135 identification

**Figure 1:**
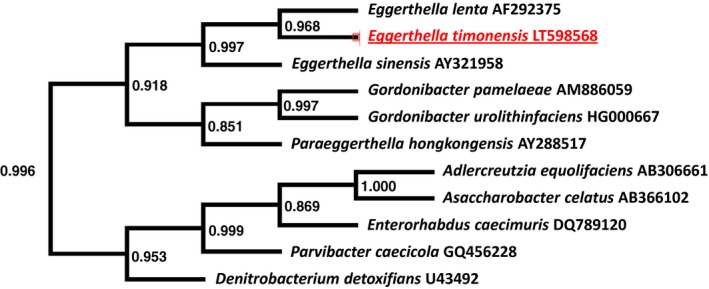
Phylogenetic tree showing the placement of *Eggerthella timonensis* strain Marseille‐P3135 relative to other close species. 16S RNA sequences are recovered after a nucleotide blast with the database of Silva project “The All‐Species Living Tree” (LTPs121). Muscle was used for sequence alignment and Fast tree for approximately maximum likelihood sequence tree generation. Local values obtained with the Shimodaira‐Hasegawa test are shown on the nodes. Duplicated and bad taxonomic reference species were removed

After comparing the 16S rRNA gene sequence of the present strain with other organisms, it was found that it exhibited a sequence similarity of 96.95% with *E. lenta* (DSM 2243; AF292375), its phylogenetically closest species with standing in nomenclature (Figure 1). The phylogenetic analysis clearly supports that the studied strain is a member of the *Eggerthella* genus. Having more than 1.3% sequence divergence with its closest species, we can suggest that the isolate represents a new species named *E. timonensis* (Bilen et al., 2016). Typical spectrum obtained by MALDI‐TOF MS of the strain can be found in supplementary Figure 1.

### 3.2 Phenotypical and biochemical analysis of strain Marseille‐P3135

**Figure 2:**
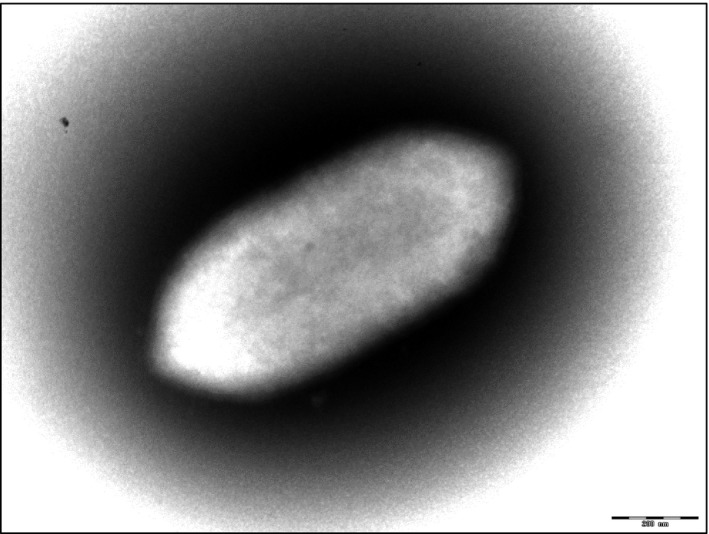
Electron micrographs of *Eggerthella timonensis* strain Marseille‐P3135 generate wit Morgagni 268D (Philips) transmission electron microscope operated at 80 keV. Scale bar = 200 nm

The strain is a nonmotile Gram‐positive rod, unable to sporulate, oxidase negative, and catalase positive. It grows under anaerobic conditions between 25°C and 42°C but optimally at 37°C. As for acidity tolerance, this strain was able to survive in media with pH ranging between 6 and 8.5 and could sustain only a 5 g/L NaCl concentration. Colonies have a smooth appearance with a mean diameter of 0.5 mm. Moreover, cells had a length of 0.7–1.6 μm when seen under electron microscope and an average diameter of 0.4 μm (Figure 2).

**Figure 3:**
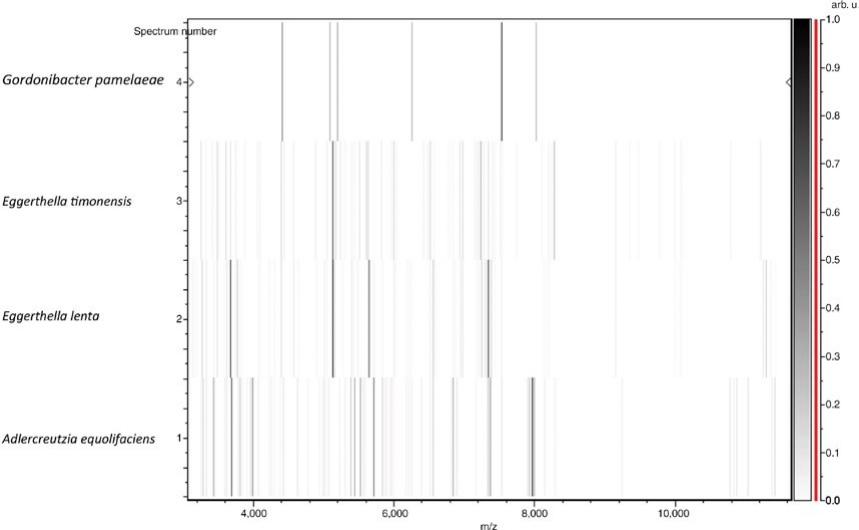
Gel view comparing mass spectra of *Eggerthella timonensis* strain Marseille‐P3135 to other species by displaying the raw spectra of different species in a pseudo‐gel like arrangement. The *x*‐axis represents the *m*/*z* value and the left *y*‐axis correspond to the running spectrum number deriving from subsequent spectra loading. Intensities of the peaks are represented by with a gray scale. Also, the correlation between the peak color and its intensity is represented in the right *y*‐axis with arbitrary units. Species shown for this analysis are noted on the left

Gel view comparing the mass spectrum of the strain and other closely related species are represented in Figure 3. We can observe that *E. lenta* and *E. timonensis* clearly presented common peaks notably around 4,550 and 6,640 m/z. *Gordonibacter pamelaeae* and *Adlercreutzia equolifaciens* which are closed of *E. timonensis* but not members of *Eggerthella* genus have a clear different spectrum profile.

Examined traits using API20A, API50CH, and APIZYM are detailed in supplementary Table 1. A comparison of some biochemical features was done in Table 1 between the studied strain and the literature data of closely related species (Lau et al., 2004b; Würdemann et al., 2009).

**Table 1:** Differential characteristics of *Eggerthella timonensis* strain Marseille‐P3135 with *Eggerthella lenta* strain NCTC 11813 (Kageyama et al., 1999), *Eggerthella sinensis* (Lau et al., 2004b), and *Gordonibacter pamelaeae* strain 7‐10‐1‐b (T) (Würdemann et al., 2009)

Properties	*E. timonensis*	*E. lenta*	*E. sinensis*	*G. pamelaeae*
Cell length (μm)	0.7–1.6	1.0–6.0	0.79	1.01 ± 0.21
Gram stain	Positive	Positive	Positive	Positive
Indole	−	−	−	Na
Endospore formation	−	−	−	−
Oxygen requirement	Strictly anaerobic	Strictly anaerobic	Strictly anaerobic	Strictly anaerobic
Major fatty acid	9‐Octadecenoic acid	12‐methyl tetradecanoic acid	Na	12‐methyl tetradecanoic acid
Motility	−	−	−	−
*Acid from:*				
D‐Ribose	+	Na	Na	Na
L‐Arabinose	+	−	−	Na
D‐glucose	+	Na	−	+
D‐Mannose	+	−	−	−
D‐Mannitol	+	−	Na	Na
Production of:				
β‐galactosidase	−	−	−	Na
Catalase	+	Na	+	+
Urease	−	−	−	Na
Alkaline phosphatase	−	−	−	−
Habitat	Human gut	Human gut	Blood culture	Human colon
G+C content (mol%)	65.17	64.2	Na	64

The major fatty acids content of this bacterium were 9‐Octadecenoic acid (37%), Octadecanoic acid (28%), and Hexadecanoic acid (28%) (Supplementary Table 2). As for its closely related species, *E. lenta* and *G. pamelaeae* had 12‐methyl tetradecanoic acid as its major component (Kageyama et al., 1999; Würdemann et al., 2009) (Table 1). These data allow us to suggest that the major fatty acid could be not representative to a genus.

Strain Marseille‐P3135 had a minimal inhibitory concentration (μg/ml) for vancomycin of 1.5, 0.047 for rifampicin, 1.5 for benzylpenicillin, 0.75 for amoxycillin, 0.94 for imipenem, 0.25 for tigecycline, 0.016 for erythromycin, 0.38 for minocycline, 0.125 for Teicoplanin, 64 for daptomycin, 0.19 for metronidazole, and more than 256 for ceftriaxone, colistin, and amikacin.

Composed of two scaffolds, the genome of strain Marseille‐P3135 is 3,916,897 bp long with 65.17 mol% G+C content. When analyzing the detected 3,371 predicted genes, 57 were RNAs (2 genes are 23S rRNA, 2 genes are 5S rRNA, 2 genes are 16S rRNA, and 51 genes are tRNA genes) and 3,314 were protein‐coding genes. Moreover, 2,524 genes (76.16%) were assigned by cogs or by NR blast a putative function. ORFans were found in 190 genes (5.73%) and 495 genes (14.94%) were annotated as hypothetical proteins (Table 2). Circular visualization of the species genome can be seen in supplementary Figure 2. The distribution of the genes into clusters of orthologous groups (COG) functional categories is represented in Table 3.

**Table 2:** Nucleotide content and gene count levels of the genome

** **	Number	Percent^a^
Size (bp)	3,916,897	100
Number of G + C	2,552,694	65.17
Number total of genes	3,371	100
Number total of protein genes	3,314	98.31
Number total of RNA genes	57	1.69
Number total of tRNA genes	51	1.51
Number total of RNA (5S, 16S, 23S) genes	6	0.18
Coding sequence size	3,459,399	88.32
Coding sequence gene protein size	3,446,256	87.98
Coding sequence tRNA gene size	3,987	0.10
Coding sequence (5S, 16S, 23S) gene size	9,156	0.23
Number of protein‐coding gene	3,314	100
Number of protein associated to COGs	2,046	61.74
Number of protein associated to orfan	190	5.73
Number of protein with peptide signal	492	14.85
Number of gene associated to resistance genes	0	0
Number of gene associated to PKS or NRPS	14	0.42
Number of genes associated to virulence	555	16.75
Number of protein with TMH	967	29.18

COG, clusters of orthologous groups.

a The total is considered either the genome size or the number of protein‐coding genes found after annotating the genome.

**Table 3:** Number and percentage of genes correlated with some COG functional categories

Code	Value	% of total	Description
[J]	167	5.04	Translation
[A]	17	0.51	Cell motility
[K]	190	5.73	Amino acid transport and metabolism
[L]	98	2.96	Cell wall/membrane biogenesis
[B]	70	2.11	Defense mechanisms
[D]	0	0	Nuclear structure
[Y]	191	5.76	Transcription
[V]	0	0	RNA processing and modification
[T]	0	0	Chromatin structure and dynamics
[M]	370	11.16	Energy production and conversion
[N]	114	3.44	Posttanslational modification, protein turnover, chaperones
[Z]	99	2.99	Signal transduction mechanisms
[W]	25	0.75	Cell cycle control, mitosis, and meiosis
[U]	34	1.03	Intracellular trafficking and secretion
[O]	79	2.38	Replication, recombination, and repair
[X]	20	0.60	Extracellular structures
[C]	0	0	Cytoskeleton
[G]	97	2.93	Coenzyme transport and metabolism
[E]	94	2.84	Carbohydrate transport and metabolism
[F]	105	3.17	Lipid transport and metabolism
[H]	10	0.30	Mobilome: prophages, transposons
[I]	65	1.96	Nucleotide transport and metabolism
[P]	175	5.28	General function prediction only
[Q]	45	1.36	Secondary metabolites biosynthesis, transport, and catabolism
[R]	105	3.17	Function unknown
[S]	125	3.77	Inorganic ion transport and metabolism
_	1.268	38.26	Not in COGs

COG, clusters of orthologous groups.

### 3.3 Comparison of genome properties

The draft genome sequence of the present new species was compared to with *G. pamelaeae* (FP929047) which is close but outside the *Eggerthella* genus and *E. lenta* (ABTT00000000) as the closest species and alone member of the genus for which the genome is available. The draft genome sequence of our strain was larger than that of *G. pamelaeae* and *E. lenta* (3,608 and 3,632 Mb, respectively). The G+C content was larger too (64% and 64.2%, respectively). The gene content was larger than that of *G. pamelaeae* and *E. lenta* (2,027 and 3,070, respectively). The functional classes’ distribution of predicted genes of the present genome according to the COGs of proteins is shown in Figure S3. The latter showed an identical profile for the three compared strains.

*E. timonensis* shared higher number of proteins with *E. lenta* (80.76%) (Table 4).

**Table 4:** This table represents in its upper right the total number of shared orthologous proteins along with the percent of similarity of the nucleotides corresponding to it in the lower left

	ET	GP	EL	DD	EC	AE
ET	**3,314**	902	1,543	937	1,164	1,204
GP	65.13	**2,027**	901	486	613	619
EL	70.56	65.79	**3,07**	942	1,146	1,19
DD	62.43	61.34	62.56	**1,762**	864	890
EC	63.23	63.83	64.36	62.04	**2,455**	1,15
AE	62.78	63.68	64.32	62.0	80.76	**2,281**

ET, *Eggerthella timonensis*; GP*, Gordonibacter pamelaeae*; EL*, Eggerthella lenta*; DD, *Denitrobacterium detoxificans*; EC, *Enterorhabdus caecimuris*; AE, *Adlercreutzia equolifaciens*.

Values with bold font represent the numbers of proteins per genome.

Subsequently, DNA–DNA hybridization values between *E. timonensis* and other species with standing in nomenclature was of 43.6 with *E. lenta*, 21.2 with *G. pamelaeae* (Table 5). Interestingly, these data show that the genome of the strain was closer than *E. lenta* one and further of *G. pamelaeae* supporting the hypothesis that strain Marseille‐P3135 as a unique species which is close to species of the *Eggerthella* genus (Kim et al., 2014; Tindall, Rosselló‐Móra, Busse, Ludwig, & Kämpfer, 2010; Wayne et al., 1987).

**Table 5:** Pairwise comparison of strain Marseille‐P3135 with other species. GGDC formula 2 was used: DDH estimates based on identities/HSP length)^a^

	ET	EL	GP	AE	EC	DD
ET	100%	43.6 [40.3%–47.1%]	21.2 [18%–24.9%]	15.1 [12.2%–18.6%]	15.1 [12.2%–18.6%]	13.2 [10.5%–16.5%]
EL		100%	22.3 [19%–25.9%]	15.3 [12.4%–18.7%]	15 [12.1%–18.4%]	13.4 [10.7%–16.7%]
GP			100%	15 [12.1%–18.4%]	15 [12.2%–18.5%]	13.3 [10.6%–16.6%]
AE				100%	26.1 [22.8%–29.7%]	13.4 [10.6%–16.7%]
EC					100%	13.4 [10.7%–16.8%]
DD						100%

ET, *Eggerthella timonensis*; GP, *Gordonibacter pamelaeae*; EL, *Eggerthella lenta*; DD, *Denitrobacterium detoxificans*; EC, *Enterorhabdus caecimuris*; AE, *Adlercreutzia equolifaciens*.

a Inherent uncertainty in DDH values estimation are represented in the confidence intervals.

## 4 CONCLUSION

In conclusion, culturomics helped us in the isolation of a new species previously uncultured from the human gut normal flora and its description using a taxonogenomics approach. Given its 16S rRNA gene sequence divergence higher than 1.3% with its phylogenetically closest species with standing in nomenclature, we propose a new species *E. timonensis*, type strain Marseille‐P3135 (=CSUR P3135, =CCUG 70327).

### 4.1 *E. timonensis* sp. nov. description

*E. timonensis (*ti.mo.nen′sis, N.L. fem. adj., with *timonensis* referring to La Timone, which is the name of the hospital in Marseille (France) where this work was performed).

The strain Marseille‐P3135^T^ (=CSUR P3135, =CCUG 70327) is the type strain of the species *E. timonensis*.

It is a nonmotile Gram‐positive rod, unable to sporulate, oxidase negative, and catalase positive. It grows under anaerobic conditions optimally at 37°C. Colonies have a smooth appearance with a mean diameter of 0.5 mm. Moreover, cells had a length of 0.7–1.6 μm when seen under electron microscope and an average diameter of 0.4 μm.

It is able to produce esterase C4, esterase lipase C8, acid phosphatase, and naphtol‐AS‐BI‐phosphohydrolase. As well, it can ferment D‐galactose, glycerol, D‐ribose, L‐arabinose, D‐xylose, D‐glucose, N‐acetylglucosamine, D‐fructose, D‐mannitol, D‐mannose, L‐rhamnose, D‐sorbitol, D‐cellobiose, amygdalin, methyl‐αD‐mannopyranoside, arbutin, escutin ferric citrate, salicin, D‐maltose, D‐lactose, D‐melibiose, D‐trehalose, D‐saccharose/sucrose, D‐raffinose, D‐melezitose, amidon, gentiobiose, D‐tagatose, D‐arabitol, L‐fucose, potassium 5‐Ketogluconate, and potassium 2‐Ketogluconate. Finally, it can ferment glucose, manitol, saccharose, lactose, maltose, xylose, salicin, arabinose, mannose, cellobiose, trehalose, and rhamnose.

The draft genome of the type strain is 3,916,897 bp long with a G + C content of 65.17%. The 16S rRNA gene and genome sequences were deposited in EMBL‐EBI under accession number LT598568 and FXXA00000000, respectively. *E. timonensis* strain Marseille‐P3135 was isolated from the stool samples of a healthy 8‐year‐old pygmy female.

## CONFLICT OF INTEREST

None to be declared.

## References

[mbo3575-bib-0001] Bilen, M. , Cadoret, F. , Daoud, Z. , Fournier, P.‐E. , & Raoult, D. (2016). “*Eggerthella timonensis*” gen. nov. isolated from a human stool sample. New Microbes and New Infections, 17, 41–42. 10.1016/j.nmni.2016.11.001 28275441 PMC5328910

[mbo3575-bib-0002] Dione, N. , Sankar, S. A. , Lagier, J.‐C. , Khelaifia, S. , Michele, C. , Armstrong, N. , … Fournier, P.‐E. (2016). Genome sequence and description of Anaerosalibacter massiliensis sp. nov. New Microbes and New Infections, 10, 66–76. 10.1016/j.nmni.2016.01.002 26937282 PMC4753391

[mbo3575-bib-0003] Eggerth, A. H. (1935). The Gram‐positive Non‐spore‐bearing Anaerobic Bacilli of Human Feces. Journal of Bacteriology, 30, 277–299. 16559837 10.1128/jb.30.3.277-299.1935 PMC543656

[mbo3575-bib-0004] Elsawi, Z. , Togo, A. H. , Beye, M. , Dubourg, G. , Andrieu, C. , Armsrtong, N. , … Khelaifia, S. (2017). Hugonella massiliensis gen. nov., sp. nov., genome sequence, and description of a new strictly anaerobic bacterium isolated from the human gut. MicrobiologyOpen, 6, e00458 10.1002/mbo3.458 PMC5552949 28326685

[mbo3575-bib-0005] Gill, S. R. , Pop, M. , DeBoy, R. T. , Eckburg, P. B. , Turnbaugh, P. J. , Samuel, B. S. , … Nelson, K. E. (2006). Metagenomic Analysis of the Human Distal Gut Microbiome. Science, 312, 1355–1359. 10.1126/science.1124234 16741115 PMC3027896

[mbo3575-bib-0006] Gouret, P. , Paganini, J. , Dainat, J. , Louati, D. , Darbo, E. , Pontarotti, P. , & Levasseur, A. (2011). Integration of evolutionary biology concepts for functional annotation and automation of complex research in evolution: The multi‐agent software system DAGOBAH In Evolutionary biology – Concepts, biodiversity, macroevolution and genome evolution (pp. 71–87). Berlin, Heidelberg: Springer 10.1007/978-3-642-20763-1_5

[mbo3575-bib-0007] Gouret, P. , Thompson, J. D. , & Pontarotti, P. (2009). PhyloPattern: Regular expressions to identify complex patterns in phylogenetic trees. BMC Bioinformatics, 10, 298 10.1186/1471-2105-10-298 19765311 PMC2759962

[mbo3575-bib-0008] Gouret, P. , Vitiello, V. , Balandraud, N. , Gilles, A. , Pontarotti, P. , & Danchin, E. G. J. (2005). FIGENIX: Intelligent automation of genomic annotation: Expertise integration in a new software platform. BMC Bioinformatics, 6, 198 10.1186/1471-2105-6-198 16083500 PMC1188056

[mbo3575-bib-0009] Greub, G. (2012). Culturomics: A new approach to study the human microbiome. Clinical Microbiology and Infection, 18, 1157–1159. 10.1111/1469-0691.12032 23148445

[mbo3575-bib-0010] Kageyama, A. , Benno, Y. , & Nakase, T. (1999). Phylogenetic evidence for the transfer of Eubacterium lentum to the genus *Eggerthella* as *Eggerthella lenta* gen. nov., comb. nov. International Journal of Systematic and Evolutionary Microbiology, 49, 1725–1732. 10.1099/00207713-49-4-1725 10555354

[mbo3575-bib-0011] Kim, M. , Oh, H.‐S. , Park, S.‐C. , & Chun, J. (2014). Towards a taxonomic coherence between average nucleotide identity and 16S rRNA gene sequence similarity for species demarcation of prokaryotes. International Journal of Systematic and Evolutionary Microbiology, 64, 346–351. 10.1099/ijs.0.059774-0 24505072

[mbo3575-bib-0012] Lagier, J.‐C. , Armougom, F. , Million, M. , Hugon, P. , Pagnier, I. , Robert, C. , … Raoult, D. (2012a). Microbial culturomics: Paradigm shift in the human gut microbiome study. Clinical Microbiology and Infection, 18, 1185–1193. 10.1111/1469-0691.12023 23033984

[mbo3575-bib-0013] Lagier, J.‐C. , Edouard, S. , Pagnier, I. , Mediannikov, O. , Drancourt, M. , & Raoult, D. (2015). Current and past strategies for bacterial culture in clinical microbiology. Clinical Microbiology Reviews, 28, 208–236. 10.1128/CMR.00110-14 25567228 PMC4284306

[mbo3575-bib-0014] Lagier, J.‐C. , El Karkouri, K. , Nguyen, T.‐T. , Armougom, F. , Raoult, D. , & Fournier, P.‐E. (2012b). Non‐contiguous finished genome sequence and description of *Anaerococcus senegalensis* sp. nov. Standards in Genomic Sciences, 6, 116–125. 10.4056/sigs.2415480 22675604 PMC3359877

[mbo3575-bib-0015] Lagier, J.‐C. , Khelaifia, S. , Alou, M. T. , Ndongo, S. , Dione, N. , Hugon, P. , … Raoult, D. (2016). Culture of previously uncultured members of the human gut microbiota by culturomics. Nature Microbiology, 1, 16203 10.1038/nmicrobiol.2016.203 PMC12094094 27819657

[mbo3575-bib-0016] Lau, S. K. P. , Woo, P. C. Y. , Fung, A. M. Y. , Chan, K. , Woo, G. K. S. , & Yuen, K. (2004a). Anaerobic, non‐sporulating, Gram‐positive bacilli bacteraemia characterized by 16S rRNA gene sequencing. Journal of Medical Microbiology, 53, 1247–1253. 10.1099/jmm.0.45803-0 15585505

[mbo3575-bib-0017] Lau, S. K. P. , Woo, P. C. Y. , Woo, G. K. S. , Fung, A. M. Y. , Wong, M. K. M. , Chan, K. , … Yuen, K. (2004b). Eggerthella hongkongensis sp. nov. and eggerthella sinensis sp. nov., two novel Eggerthella species, account for half of the cases of Eggerthella bacteremia. Diagnostic Microbiology and Infectious Disease, 49, 255–263. 10.1016/j.diagmicrobio.2004.04.012 15313530

[mbo3575-bib-0018] Ley, R. E. , Bäckhed, F. , Turnbaugh, P. , Lozupone, C. A. , Knight, R. D. , & Gordon, J. I. (2005). Obesity alters gut microbial ecology. Proceedings of the National Academy of Sciences of the United States of America, 102, 11070–11075. 10.1073/pnas.0504978102 16033867 PMC1176910

[mbo3575-bib-0019] Ley, R. E. , Turnbaugh, P. J. , Klein, S. , & Gordon, J. I. (2006). Microbial ecology: Human gut microbes associated with obesity. Nature, 444, 1022–1023. 10.1038/4441022a 17183309

[mbo3575-bib-0020] Moore, W. E. C. , Cato, E. P. , & Holdeman, L. V. (1971). Eubacterium lentum (Eggerth) Prévot 1938: Emendation of Description and Designation of the Neotype Strain. International Journal of Systematic and Evolutionary Microbiology, 21, 299–303. 10.1099/00207713-21-4-299

[mbo3575-bib-0021] Morel, A.‐S. , Dubourg, G. , Prudent, E. , Edouard, S. , Gouriet, F. , Casalta, J.‐P. , … Raoult, D. (2015). Complementarity between targeted real‐time specific PCR and conventional broad‐range 16S rDNA PCR in the syndrome‐driven diagnosis of infectious diseases. European Journal of Clinical Microbiology & Infectious Diseases, 34, 561–570. 10.1007/s10096-014-2263-z 25348607

[mbo3575-bib-0022] Price, M. N. , Dehal, P. S. , & Arkin, A. P. (2009). FastTree: Computing large minimum evolution trees with profiles instead of a distance matrix. Molecular Biology and Evolution, 26, 1641–1650. 10.1093/molbev/msp077 19377059 PMC2693737

[mbo3575-bib-0023] Raoult, D. , & Henrissat, B. (2014). Are stool samples suitable for studying the link between gut microbiota and obesity? European Journal of Epidemiology, 29, 307–309. 10.1007/s10654-014-9905-4 24838696

[mbo3575-bib-0024] Seng, P. , Drancourt, M. , Gouriet, F. , La Scola, B. , Fournier, P.‐E. , Rolain, J. M. , & Raoult, D. (2009). Ongoing revolution in bacteriology: Routine identification of bacteria by matrix‐assisted laser desorption ionization time‐of‐flight mass spectrometry. Clinical Infectious Diseases, 49, 543–551. 10.1086/600885 19583519

[mbo3575-bib-0025] The SILVA and “All‐species Living Tree Project (LTP)” taxonomic frameworks [WWW Document] (n.d.) Retrieved from https://www.ncbi.nlm.nih.gov/pmc/articles/PMC3965112/ (accessed 7.10.17).

[mbo3575-bib-0026] Tindall, B. J. , Rosselló‐Móra, R. , Busse, H.‐J. , Ludwig, W. , & Kämpfer, P. (2010). Notes on the characterization of prokaryote strains for taxonomic purposes. International Journal of Systematic and Evolutionary Microbiology, 60, 249–266. 10.1099/ijs.0.016949-0 19700448

[mbo3575-bib-0027] Wayne, L. G. , Brenner, D. J. , Colwell, R. R. , Grimont, P. A. D. , Kandler, O. , Krichevsky, M. I. , … Truper, H. G. (1987). Report of the Ad Hoc Committee on Reconciliation of Approaches to Bacterial Systematics. International Journal of Systematic and Evolutionary Microbiology, 37, 463–464. 10.1099/00207713-37-4-463

[mbo3575-bib-0028] Würdemann, D. , Tindall, B. J. , Pukall, R. , Lünsdorf, H. , Strömpl, C. , Namuth, T. , … Oxley, A. P. A. (2009). *Gordonibacter pamelaeae* gen. nov., sp. nov., a new member of the Coriobacteriaceae isolated from a patient with Crohn's disease, and reclassification of *Eggerthella hongkongensis* Lau et al. 2006 as *Paraeggerthella hongkongensis* gen. nov., comb. nov. International Journal of Systematic and Evolutionary Microbiology, 59, 1405–1415. 10.1099/ijs.0.005900-0 19502325

