# Supplementary figures and images for: Eggerthella timonensis sp. nov, a new species isolated from the stool sample of a pygmy female
Source: Microbiologyopen. 2018 Jun 13;7(5):e00575. doi: 10.1002/mbo3.575 (PMC6182555; doi:10.1002/mbo3.575)

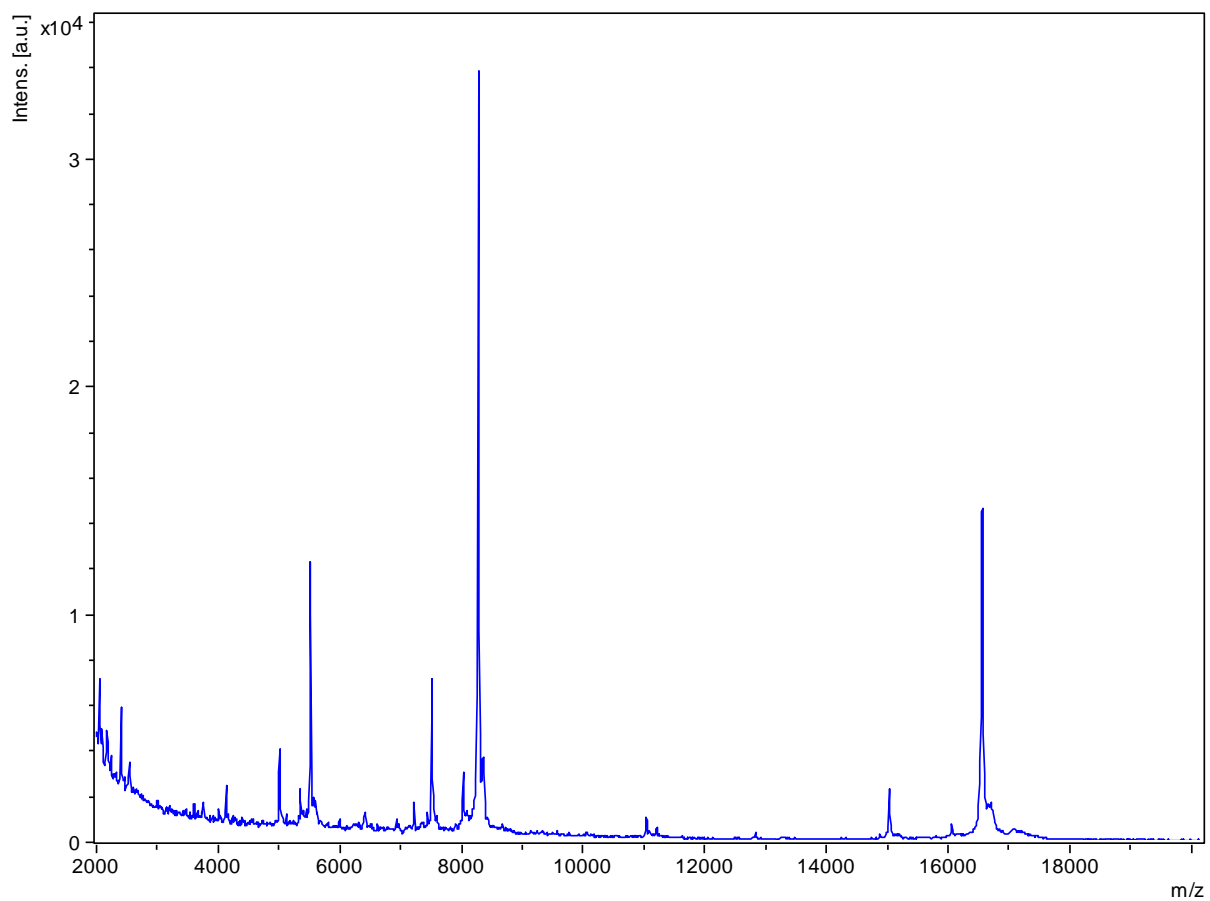

Supplement: Supplementary file 1 [file MBO3-7-e00575-s001.pdf]

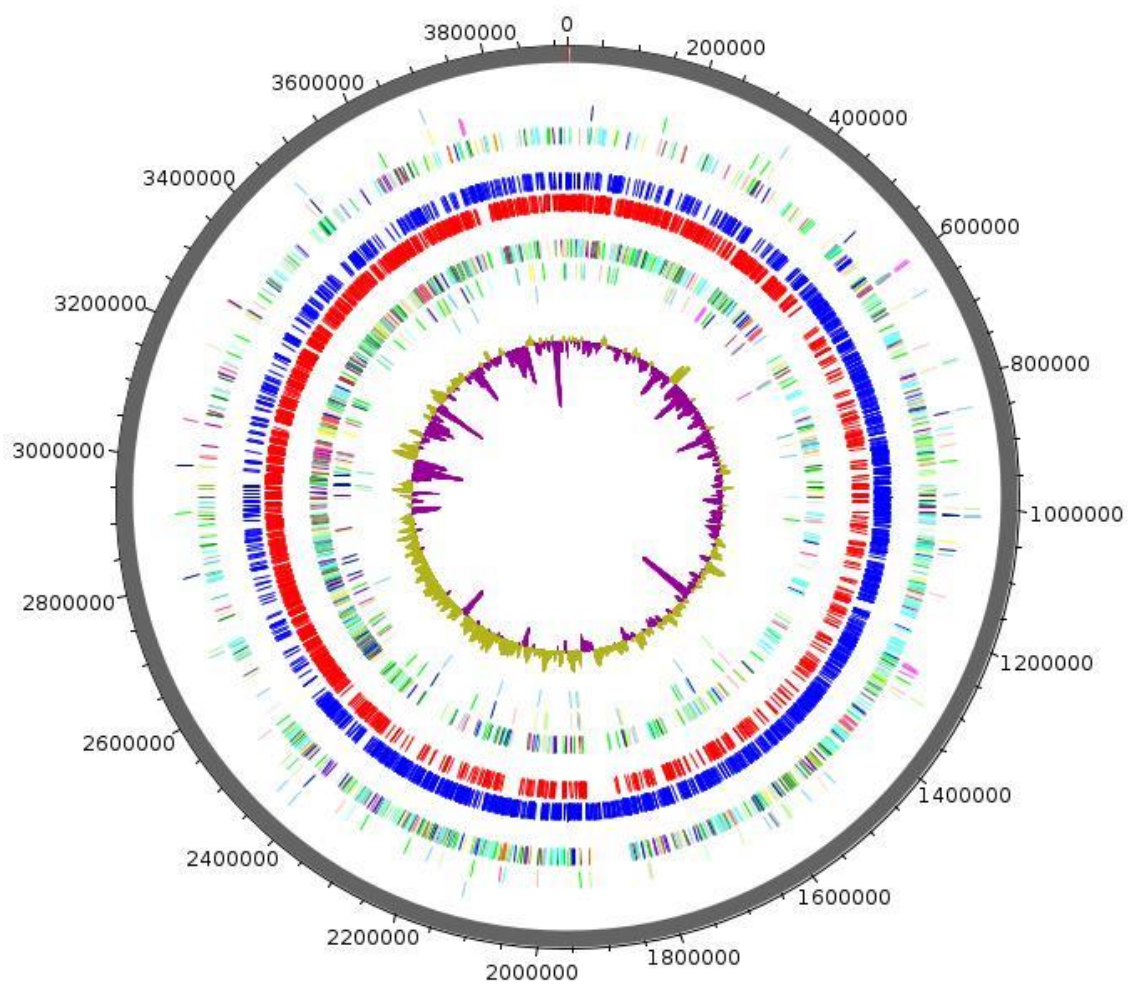

Supplement: Supplementary file 2 [file MBO3-7-e00575-s002.pdf]
