# Supplementary material for: Eggerthella timonensis sp. nov, a new species isolated from the stool sample of a pygmy female
Source: Microbiologyopen. 2018 Jun 13;7(5):e00575. doi: 10.1002/mbo3.575 (PMC6182555; doi:10.1002/mbo3.575)
Supplement: Supplementary file 3 [file MBO3-7-e00575-s003.pdf]

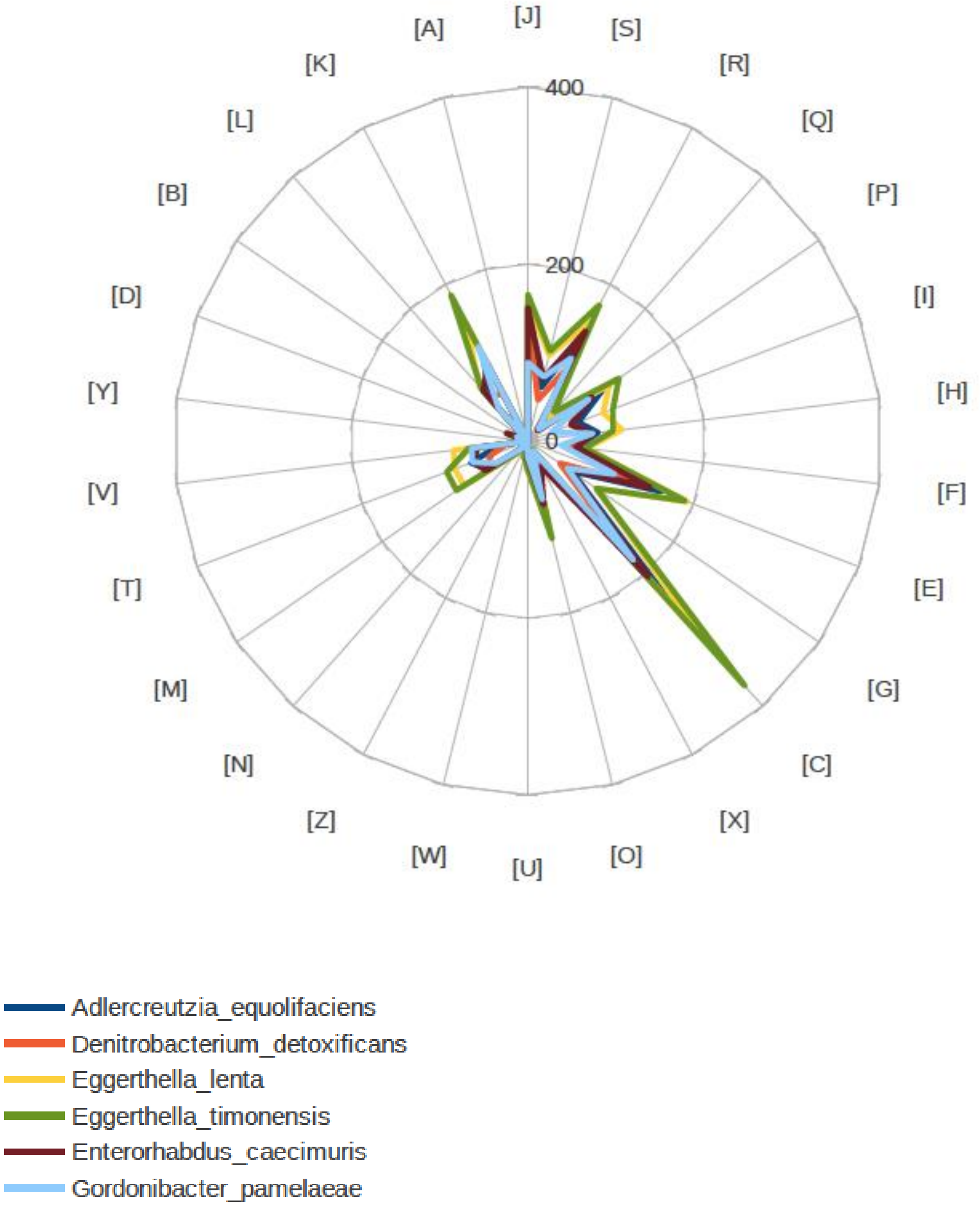
[J]
[S]
[R]
[Q]
[P]
[I]
[H]
[F]
[E]
[G]
[C]
[X]
[O]
[U]
[W]
[Z]
[N]
[M]
[T]
[V]
[Y]
[D]
[B]
[L]
[K]
[A]
400
200
0
Adlercreutzia_equolifaciens
Denitrobacterium_detoxificans
Eggerthella_lenta
Eggerthella_timonensis
Enterorhabdus_caecimuris
Gordonibacter_pamelaeae
