# Supplementary material for: Eggerthella timonensis sp. nov, a new species isolated from the stool sample of a pygmy female
Source: Microbiologyopen. 2018 Jun 13;7(5):e00575. doi: 10.1002/mbo3.575 (PMC6182555; doi:10.1002/mbo3.575)
Supplement: Supplementary file 4 [file MBO3-7-e00575-s004.docx]

| **Table S1. API results for strain Marseille-P3135** | | | | |  |  |
| --- | --- | --- | --- | --- | --- | --- |
|  | | **+/-** |  | | **+/-** |  |
| Alkaline phosphatase | | - | D-mannitol | | + |  |
| Esterase Lipase (C8) | | + | D-trehalose | | + |  |
| Esterase (C4) | | + | D-saccharose | | + |  |
| Leucine arylamidase | | - | ARButine | | + |  |
| Lipase (C14) | | - | D-Maltose | | + |  |
| Valine arylamidase | | - | AMYgdaline | | + |  |
| Naphtol-AS-BI-phosphohydrolase | | + | D-rafinose | | + |  |
| N-acetyl-β-glucosaminidase | | - | D-melibiose | | + |  |
| acid phosphatase | | + | ESCutine | | + |  |
| Cystine arylamidase | | - | D-cellobiose | | + |  |
| α-glucosidase | | - | xylitol | | - |  |
| β-glucuronidase | | - | D-lactose | | + |  |
| α-galactosidase | | - | Inuline | | - |  |
| α-chymotrypsin | | - | Amidon | | + |  |
| α-mannosidase | | - | Potassium 2-Cetogluconate | | + |  |
| β-glucosidase | | - | D-tagatose | | + |  |
| β-galactosidase | | - | D-turanose | | - |  |
| Trypsin | | - | D-lyxose | | - |  |
| α-fucosidase | | - | D-arabitol | | + |  |
| Glycerol | | + | L-fucose | | + |  |
| L-arabinose | | + | Potassium 5-Cetogluconate | | + |  |
| D-adonitol | | - | L-arabitol | | - |  |
| Inositol | | - | D-Fucose | | - |  |
| Salicine | | + | Potassium gluconate | | - |  |
| Methyl-αD-glucosamine | | - | Indole formation | | - |  |
| L-xylose | | - | UREase | | - |  |
| D-melezitose | | + | Acidication (GlUcose) | | + |  |
| N-acetylglucosamine | | + | Acidification (MANitol) | | + |  |
| Methul-αD-mannopyranoside | | + | Acidification (LACtose) | | + |  |
| D-arabinose | | - | Acidification (SACcharose) | | + |  |
| L-sorbose | | - | Acidificatiom (MALtose) | | + |  |
| D-glucose | | + | Acidificatiom (SALicin) | | + |  |
| D-galactose | | + | Acidification (XYLose) | | + |  |
| Glucogene | | - | Acidifiction (ARAbinose) | | + |  |
| D-ULcitol | | - | Hydrolysis (protease) (GELatin) | | + |  |
| Gentiobiose | | + | Hydrolysis β- glucosidase (ESCulin) | | - |  |
| D-mannose | | + | acidification (GLYcerol) | | - |  |
| D-sorbitol | | + | Acidification (CELlobiose) | | + |  |
| D-ribose | | + | Acidification ManNosE) | | + |  |
| D-fructose | | + | Acidicatiom (MeLeZitose) | | - |  |
| D-xylose | | + | Acidification (RaFfinose) | | - |  |
| L-rhamnose | | + | Acidication (SORbitol) | | - |  |
| Erythritol | | - | Acidification (RHAmnose) | | + |  |
| Methyl-βD-xylopyranoside | | - | Acidification (TREhalose) | | + |  |
| **Table S2.** Cellular fatty acids composition of strain Marseille-P3135. | | | |  | | |
| **Fatty acids** | **Name** | | | **Mean relative % (a)** | | |
| 18:1n9 | 9-Octadecenoic acid | | | 36.9 ± 7.7 | | |
| 18:00 | Octadecanoic acid | | | 28.0 ± 7.7 | | |
| 16:00 | Hexadecanoic acid | | | 28.0 ± 0.8 | | |
| 18:1n7 | 11-Octadecenoic acid | | | 2.6 ± 1.3 | | |
| 18:2n6 | 9,12-Octadecadienoic acid | | | 2.3 ± 0.8 | | |
| 14:00 | Tetradecanoic acid | | | 2.2 ± 1.2 | | |
| ^a^ Mean peak area percentage ; TR = trace amounts < 1 % | | | |  | | |
